# Supplementary material for: Analysis of Predictive Factors for Successful Vascular Anastomoses in a Sheep Uterine Transplantation Model
Source: J Clin Med. 2022 Sep 6;11(18):5262. doi: 10.3390/jcm11185262 (PMC9503062; doi:10.3390/jcm11185262)
Supplement: Supplementary file 1 [file jcm-11-05262-s001.zip › jcm-1802964-supplementary.pdf]

Supplementary Materials: The following supporting information can be downloaded at: www.mdpi.com/xxx/s1, Table S1: Predictive factors for success of an arterial anastomosis at D4; Table S2: Predictive factors for success of an venous anastomosis at D4. Table S3: Comparison of lactate levels at different times according to short-term success or failure.

Table S1. Predictive factors for success of an arterial anastomosis at D4.

**Variable**	**Succes N = 17 (65.4%)**		**Failure N = 9 (34.6%)**		**p-Value**
**Ewe characteristic**					
Age (month)	37.5 ± 2.8	33.4 - 44.6	39.4 ± 6.6	33.1 – 49.1	0.17
Weight	69.9 ± 10.8	57.0 – 92.0	67.8 ± 17.4	55.0 – 97.0	**0.03**
**Anatomical data**					
Caliber (mm)	5.3 ± 2.3	2.5 – 10.0	4.6 ± 2.2	1.0 – 8.0	0.5
Length (mm)	87.4 ± 19.9	60 – 120	76.7 ± 10.9	65 - 100	0.15
**Transplantation**					
Operating time (min)	415.9 ± 29.4	377 – 450	454.2 ± 51.3	385 - 516	**0.02**
Time of uterine dissection	74.9 ± 12.7	58 – 103	70.4 ± 14.4	53 - 92	0.42
Cold ischemia	43.1 ± 7.7	33 – 60	49.6 ± 7.6	41 – 60	**0.05**
< 40 min	6	35.3	0	0.0	**0.04**
> 40 min	11	64.7	9	100.0	
Warm ischemia	90.3 ± 12.3	75 – 113	107.7 ± 28.2	87 - 156	**0.04**
< 120 min	17	100.0	7	77.8	**0.04**
> 120 min	0	0.0	2	22.2	
**Anastomoses**					
Anastomoses time	18.4 ± 5.5	10 - 31	25.8 ± 19.6	8 - 76	0.1
Posterior branch					
Yes	16	94.1	16	94.1	0.63
No	1	5.9	1	11.1	
Papaverine					
Yes	1	5.9	2	22.2	0.21
No	16	94.1	7	77.8	
Anastomoses complications					
Yes	0	0.0	2	22.2	**0.04**
No	17	100.0	7	77.8	
Remedial points					
Yes	5	29.4	4	44.4	0.44
No	12	70.6	5	55.6	
Reverse Flow					
Yes	9	52.9	3	33.3	0.34
No	8	47.1	6	66.7	

Table S2. Predictive factors for success of an venous anastomosis at D4.

**Variable**	**Succes N =21 (84%)**		**Failure N = 4 (16%)**		**p-Value**
**Ewe characteristic**					
Age (month)	36.5 ± 2.4	33.1 - 40.6	43.9 ± 4.8	37.4 – 49.1	0.17
Weight	67.6 ± 11.1	55.0 – 92.0	70.5 ± 18.3	55.0 – 97.0	**0.03**
**Anatomical data**					
Caliber (mm)	5.6 ± 2.4	2.0 – 10.0	5.3 ± 2.1	3.0 – 8.0	0.79
Length (mm)	81.2 ± 17.7	50 – 120	75.0 ± 30.3	55 - 120	0.57

**Transplantation**					
Operating time (min)	417.9 ± 33.2	377 - 490	466.5 ± 43.5	430 - 516	**0.02**
Time of uterine dissection	75.7 ± 11.7	58 - 103	66.3 ± 17.5	53 - 92	0.18
Cold ischemia	42.9 ± 6.2	33 - 51	54.8 ± 9.2	41 – 60	**0.003**
< 40 min	6	28.6	0	0.0	0.22
> 40 min	15	71.4	4	100.0	
Warm ischemia	93.8 ± 18.2	75 – 156	105.3 ± 33.8	88 - 156	0.33
< 120 min	20	95.2	3	75.0	0.17
> 120 min	1	4.8	1	25.0	
**Anastomoses**					
Anastomoses time	22.3 ± 4.6	15 - 35	27.5 ± 7.1	20 - 37	**0.07**
Anastomoses complications					
Yes	0	0.0	1	25.0	**0.02**
No	21	100.0	3	75.0	
Remedial points					
Yes	2	9.5	1	25.0	0.38
No	19	90.5	3	75.0	

Table S3. Comparison of lactate levels at different times according to short-term success or failure.

**Lactates levels**	**Success N = 7**		**Failure N = 5**		**p-Value**
	Mean ± SD	Range	Mean ± SD	Range	
**T0**	2.04 ± 1.05	0.80 – 3.48	1.44 ± 0.35	0.82 – 1.68	0.25
**T1**	1.10 ± 0.34	0.56 – 1.53	1.07 ± 0.40	0.42 – 1.36	0.91
**T2**	2.48 ± 0.85	1.48 – 4.00	4.60 ± 2.44	1.75 – 7.43	0.06
**T3**	1.00 ± 0.18	0.76 – 1.21	2.20 ± 2.00	0.86 – 5.71	0.14
**T4**	0.97 ± 0.6	0.43 – 1.83	1.87 ± 2.49	0.30 – 5.57	0.37
**T5**	1.06 ± 0.76	0.45 – 2.62	1.06 ± 0.88	0.30 – 2.24	0.99
